# Editorial: Reviews in neonatology 2024

**DOI:** 10.3389/fped.2025.1619870

**Published:** 2025-05-14

**Authors:** Thangaraj Abiramalatha, Giuseppe De Bernardo

**Affiliations:** ^1^ Kovai Medical Center and Hospital (KMCH), KMCH Research Foundation, Coimbatore, India; ^2^ Department of Woman and Child, Buon Consiglio Fatebenefratelli Hospital, Naples, Italy

**Keywords:** preterm newborn, neonatal care, NEC, BPD, SIP, respiratory distress

**Editorial on the Research Topic**
Reviews in neonatology 2024

The management of premature neonates remains a major focus in neonatal care and research. Current special issue address key aspects such as the prevention of bronchopulmonary dysplasia (BPD), the optimal use of ventilation, and the timing of hydrocortisone in neonatal shock. Other important areas include the safety of pharmacologic treatments like non-steroidal anti-inflammatory drugs (NSAIDs) and acetaminophen, particularly in relation to spontaneous intestinal perforations, and the quality of essential newborn care provided by healthcare workers in low-resource settings.

The systematic review and meta-analysis performed by Ramaswamy et al. evaluates the impact of early vs. late hydrocortisone administration in neonates with shock requiring vasopressor support. Based on 20 studies, early initiation—especially when inotropic support begins—may improve treatment response and reduce inotrope duration, with no major adverse effects. In contrast, late hydrocortisone use is potentially associated with higher risks of mortality, necrotizing enterocolitis (NEC), and longer hospital stays. The evidence quality was mostly very low, leading to cautious recommendations. The guideline suggests considering hydrocortisone when dopamine dosage reaches ≥10 µg/kg/min. Despite variability in dosing and populations across studies, early hydrocortisone appears beneficial.

A systematic review and network meta-analysis conducted by Yang et al. evaluates the efficacy and safety of various noninvasive ventilation (NIV) strategies following extubation in neonates with Neonatal Respiratory Distress Syndrome (NRDS). Analyzing data from 23 randomized controlled trials involving 2,331 neonates, the study compares four NIV modalities: nasal continuous positive airway pressure (NCPAP), non-invasive intermittent positive pressure ventilation (NIPPV), nasal bi-level positive airway pressure (N-BiPAP), and non-invasive high-frequency oscillatory ventilation (NHFOV). Results show NHFOV as the most effective approach, significantly reducing reintubation rates and carbon dioxide retention compared to NCPAP, NIPPV, and N-BiPAP. NHFOV and NIPPV also demonstrated a notable reduction in bronchopulmonary dysplasia (BPD) incidence. No significant differences were observed among modalities in terms of nasal injury, air leaks, intraventricular hemorrhage, or mortality, suggesting comparable safety profiles. The findings support NHFOV as the optimal choice for post-extubation respiratory support in preterm infants with NRDS.

Dini et al. reported that BPD is a major complication of prematurity, affecting especially extremely low birth weight and gestational age infants. This mini-review provides a comprehensive overview of current strategies aimed at preventing BPD, a condition arising from disrupted lung development and compounded by invasive respiratory support and oxygen toxicity. Advances in neonatal care—such as antenatal corticosteroids, early surfactant administration via minimally invasive techniques (e.g., LISA), and non-invasive ventilation methods—have led to better survival rates but have not significantly reduced BPD incidence. Pharmacological interventions including caffeine, vitamin A, and selective postnatal corticosteroids show promise, particularly when used in targeted, time-sensitive approaches. Emerging therapies like stem cell treatment and azithromycin are under investigation, while certain interventions (e.g., inhaled nitric oxide and routine diuretics) remain controversial due to insufficient evidence. Nutritional support, especially human milk, plays a crucial role in lung development and inflammation modulation. The review underscores the importance of individualized, evidence-based clinical decisions, considering both risks and benefits. Ongoing research into genetic, environmental, and therapeutic factors is essential to refine prevention strategies and improve long-term outcomes for preterm infants at risk of BPD.

The question of whether NSAID use increases the risk of spontaneous intestinal perforation (SIP) is highly relevant in clinical practice. This is particularly important because the population most likely to receive NSAIDs for the closure of a patent ductus arteriosus (PDA) – the extreme preterm neonates - is also the one most vulnerable to developing SIP. A systematic review by Hudson et al. explored this potential association by evaluating the relationship between SIP and the use of NSAIDs, including indomethacin, ibuprofen, and paracetamol. An earlier systematic review by Thakkar et al. identified a significant association between postnatal indomethacin use and the risk of SIP (1). In contrast, the review by Hudson et al. did not find a significant association with indomethacin, nor with ibuprofen or acetaminophen. In light of these conflicting results, the authors emphasize the need for additional high-quality studies to evaluate the association between NSAID use and the development of SIP.

Essential Newborn Care (ENC), as recommended by the World Health Organization (WHO), is a vital component of neonatal health care. Evidence from low- and middle-income countries (LMICs) consistently shows that the implementation and adherence to ENC practices significantly lower neonatal mortality, especially in the early neonatal period (2, 3). In Ethiopia, the early neonatal mortality rate stands at 26.5 per 1,000 live births—substantially higher than in many other LMICs (4). This underscores the urgent need for effective ENC practices in the country. The systematic review by Geta Hardido et al. evaluated the knowledge and practice of ENC among Ethiopian healthcare providers, revealing concerning gaps: only 57% demonstrated adequate knowledge, and just 54% adhered to recommended practices. These findings highlight the pressing need for enhanced education and training of healthcare workers to reduce early neonatal deaths. Another systematic review from the region identified additional barriers, including transportation challenges, high costs, limited access to care, and poor maternal awareness of neonatal danger signs (5). Healthcare systems also faced issues such as insufficient training and staff retention, as well as shortages in essential supplies, standardized protocols, and data collection mechanisms. It is crucial that the Ethiopian government and relevant stakeholders act swiftly to address these systemic challenges, with the goal of strengthening ENC implementation and lowering neonatal mortality rates.

The articles presented in this special issue underscore the multifaceted challenges and evolving strategies in neonatal care, particularly for preterm and critically ill neonates. From pharmacologic interventions and respiratory support to the implementation of essential newborn care in low-resource settings, each review contributes critical insights that can inform clinical practice and policy. Although promising approaches such as early hydrocortisone use, noninvasive ventilation strategies like NHFOV, and targeted prevention of BPD show potential for improving outcomes, the variability in evidence quality highlights the continued need for high-quality, context-sensitive research. Equally important is the translation of this knowledge into practice—especially in resource-limited settings—through education, training, and health system strengthening. Together, these efforts hold the potential to advance neonatal outcomes globally and bridge the persistent gaps in care for the most vulnerable infants.
